# Elevated and Correlated Expressions of miR-24, miR-30d, miR-146a, and SFRP-4 in Human Abdominal Adipose Tissue Play a Role in Adiposity and Insulin Resistance

**DOI:** 10.1155/2018/7351902

**Published:** 2018-03-12

**Authors:** Yury O. Nunez Lopez, Gabriella Garufi, Magdalena Pasarica, Attila A. Seyhan

**Affiliations:** ^1^Translational Research Institute for Metabolism and Diabetes, Florida Hospital, Orlando, FL, USA; ^2^University of Central Florida College of Medicine, Orlando, FL, USA; ^3^Sanford Burnham Prebys Medical Discovery Institute, Orlando, FL, USA; ^4^The Chemical Engineering Department, Massachusetts Institute of Technology, Cambridge, MA, USA

## Abstract

**Objective:**

We explored the relationships among microRNAs (miRNAs) and SFRP4, as they relate to adipose tissue functions including lipolysis, glucose and glycerol turnover, and insulin sensitivity.

**Methods:**

Abdominal adipose tissue (AbdAT) levels of thirteen microRNAs (miRNAs), SFRP4, and VEGF in lean nondiabetic subjects (*n* = 7), subjects with obesity (*n* = 5), and subjects with obesity and type 2 diabetes (T2DM) (*n* = 5) were measured by qPCR. Insulin sensitivity was measured by the euglycemic-hyperinsulinemic clamp. Osmium fixation and Coulter counting were used for adipocyte sizing. Data were analyzed using generalized linear models that adjusted for age, gender, and ethnicity.

**Results:**

AbdAT miR-24, miR-30d, and miR-146a were elevated in subjects with obesity (*P* < 0.05) and T2DM (*P* < 0.1) and positively correlated with measures of percent body fat by DXA (r_miR.24_ = 0.894, r_miR.146a_ = 0.883, *P* < 0.05), and AbdAT SFRP4 (r_miR.30_ = 0.93, r_miR.146a_ = 0.88, *P* < 0.05). These three miRNAs additionally correlated among themselves (r_miR.24~miR.146a_ = 0.90, r_miR.30~miR.146a_ = 0.85, *P* < 0.01).

**Conclusions:**

This study suggests a novel association between the elevated levels of miRNAs miR-24, miR-30d, and miR-146a (apparently coregulated) and the level of SFRP4 transcript in AbdAT of subjects with obesity and T2DM. These molecules might be part of a regulatory loop involved in AbdAT remodeling/adiposity and systemic insulin resistance. This trial is registered with NCT00704197.

## 1. Introduction

Obesity increases the risk of developing serious health complications including hypertension, steatohepatitis, fatty liver, dyslipidemia, and type 2 diabetes (T2DM) [1]. For a condition as prevalent as obesity, with eventual life-threatening complications, our knowledge of mechanisms leading to obesity-associated pathophysiological alterations is limited. Pathological adipose tissue expansion leads to massive enlargement of adipocytes, limited angiogenesis, and ensuing hypoxia [2]. In addition, obesity associates with insulin resistance (including insulin suppression of lipolysis) and increased fat cell size (FCS). However, enlarged subcutaneous abdominal adipocyte size, but not obesity itself, was shown to predict T2DM, independent of insulin resistance [3].

Earlier, we found that subcutaneous adipose tissue from humans with obesity has inadequate vascularization, hypoxia, inflammation [4, 5], and fibrosis [6]. These alterations were found to be correlated with insulin resistance [4–10]. We have also demonstrated that the antiangiogenic factor secreted frizzled-related protein 4 (SFRP4) is associated with adipose tissue rarefaction (capillary drop out) and may lead to inflammation and ultimately insulin resistance in people with obesity [11]. Other groups demonstrated the clinical utility of circulating SFRP4 protein [12] by showing it could predict the development of T2DM up to five years before the onset of T2DM [13]. Mechanistically, SFRP4 appears to act by decreasing insulin secretion from the pancreatic beta cells [13], by preventing transcription of angiogenesis-associated genes (including vascular endothelial growth factor, VEGF) [12], and by modulating Wnt signaling (pathway involved in glucose metabolism) [14]. Concordantly, our group has shown that the dysregulation of Wnt signaling, driven by changes in the expression of a related family member (SFRP3), is also associated with inflammation, glucose metabolism, and insulin resistance in the skeletal muscle of insulin-resistant humans [15]. We have also shown the potential biomarker utility of the circulating SFRP4 protein in an independent cohort by demonstrating its differential abundance in plasma from subjects with obesity, prediabetes, and T2DM [16].

MicroRNAs (miRNAs) are endogenous, small noncoding RNAs that are abundant in many cell types and tissues including the adipose tissue [17]. miRNAs play important functions in the regulation of a broad spectrum of physiological and metabolic processes including obesity, metabolic dysfunction, diabetes, and aging, among others [18–21]. miRNAs are reported to stimulate or inhibit the differentiation of adipocytes and to regulate specific metabolic and endocrine functions [22]. Like adipokines, miRNAs can also be secreted from fat cells into the blood circulation and function in inter tissue or organ communication in an endocrine fashion and thus may serve as markers of dysregulated adipose tissue function [23–25]. Given their role in regulating transcriptional networks, miRNAs in adipose tissue might offer attractive biomarkers of adiposity as well as potential therapeutic targets for treating metabolic disorders.

The goal of this study was to explore relationships among miRNAs and SFRP4, as they relate to adipose tissue functions including lipolysis, glucose and glycerol turnover, and insulin sensitivity. We hypothesize that miRNAs might be involved in the regulation of SFRP4 causing adipose tissue rarefaction and inflammation and ultimately leading to insulin resistance in patients with obesity. To test this hypothesis, we conducted a pilot retrospective cross-sectional study to evaluate the relationship between AbdAT miRNAs, SFRP4, and related adipose tissue phenotypes and functions.

## 2. Methods

The parent clinical trial was conducted at Pennington Biomedical Research Center (PBRC) and the main study results previously described by Pasarica et al. [4–6]. Subjects were excluded for previous use of thiazolidinediones or drugs known to affect lipid metabolism or body weight. Subjects with T2DM were treated with lifestyle modifications, metformin, or glipizide. The protocol was approved by the PBRC Institutional Review Board and all subjects gave written informed consent. In this secondary study, 7 lean [ND mass index (BMI) < 25 kg/m^2^] and 10 subjects with obesity [BMI > 30 kg/m^2^] with (*N* = 5) or without T2DM (*N* = 5) were included. One sample from each original group had been depleted; therefore, three subjects were not included in this study.

### 2.1. Clinical Measurements

Clinical measurements were conducted as described [4, 5]. Briefly, body composition was measured by dual-energy X-ray absorptiometry (DXA) on a Hologic dual-energy X-ray absorptiometer (Hologic, MA). Maximal aerobic capacity (VO2 max) was assessed using a graded treadmill test. Insulin sensitivity was measured during a euglycemic-hyperinsulinemic clamp as the mean rate of exogenous glucose infusion during the 30 minutes steady-state, corrected for changes in glycemia and divided by fat-free mass. Insulin suppression of lipolysis was assessed as the percent change in the rate of appearance of glycerol from the basal to insulin-stimulated state. Subcutaneous adipose tissue biopsies were obtained from the abdominal areas using a blunt-ended needle.

### 2.2. Laboratory Assays

Analysis of adipose tissue SFRP4 and VEGF mRNA and serum SFRP4 protein levels was previously described [5, 11]. VEGF and SFRP4 gene expressions were measured by qRT-PCR using TaqMan® (Applied Biosystem) and normalized to the housekeeping gene beta-actin. Mean adipocyte size was measured by osmium fixation and counting on a Coulter counter. Adipose tissue capillary density was measured on a paraffin-embedded adipose tissue by using tetramethylrhodamine isothiocyanate-conjugated lectin from *Ulex europaeus* (Sigma-Aldrich) for labeling capillaries and was previously presented [5].

### 2.3. miRNA Profiling

A panel of ten miRNAs (hsa-miR-21, hsa-miR-24, hsa-miR-29a, hsa-miR-30d, hsa-miR-34a, hsa-miR-126, hsa-miR-146a, hsa-miR-148a, hsa-miR-375, and hsa-miR-376) associated with diabetes and/or obesity and three potential endogenous control miRNAs (hsa-miR-191, hsa-miR-423, and hsa-miR-451) was profiled by qRT-PCR using TaqMan microRNA assays (Thermo Fisher, CA). These miRNAs are part of a metabolically involved miRNA panel we have designed and tested for biomarker discovery efforts at the Translational Research Institute for Metabolism and Diabetes, Florida Hospital [16, 26–28]. Briefly, total RNA was extracted using the miRNeasy mini kit (QIAGEN, Valencia, CA) according to manufacturer's instructions. Complementary cDNA was reverse transcribed from 10 ng of total RNA using a primer pool of 1 : 100 diluted TaqMan microRNA assays 5xRT and the TaqMan microRNA reverse transcription kit (Thermo Fisher), following the manufacturer's recommendations. The cDNA was then quantitatively amplified using 20x TaqMan microRNA assays and 2x TaqMan Universal Master Mix II (Thermo Fisher), on a ViiA™ 7 real-time PCR system, following manufacturer's recommendations. A panel of miRNAs associated with obesity and metabolic diseases. The qRT-PCR reactions were performed in triplicate. The −ΔCt method was used for the data analysis. The NormFinder algorithm [29] was implemented in the R programing environment and used to identify hsa-miR-191 as the best endogenous miRNA for data normalization.

### 2.4. Statistical Analysis

The data were analyzed using generalized linear models (GLMs) with gamma family and log link implemented in the R environment, using the *glm* function from the *stats* package. Each GLM modeled the −ΔCt expression levels of a specific miRNA (the outcome variable) as a function of two explanatory variables: a BMI-related variable with two levels (lean and obesity) and a diabetes-related variable with two levels (ND and T2DM) (ND: subjects without diabetes). The models additionally adjusted for potential confounding effects of age, gender, and ethnicity (also included in the GLM models as explanatory variables). General linear hypothesis testing and multiple comparisons (post hoc Tukey tests) for each variable were implemented using the *glht* function from the *multcomp* package. Plots for the visualization of the relationships between the outcome and the explanatory variable of interest (as the other explanatory variables are held constant) were generated using the *visreg* package. The *visreg* package offers the advantage of superimposing partial residuals (the adjusted values) on the visualization plots. Correlation plots were generated using the *ggplot2* package and the miRNA levels expressed as −ΔCt data. The reported partial correlations and corresponding *P* values were calculated using the *pcor.test* function from the *ppcor* package, also controlling for the potential confounding effects of age, gender, and ethnicity. Statistical significance was defined relative to a nominal two-sided 5% type 1 error rate.

## 3. Results

### 3.1. Anthropometric Characteristics

A total of 17 subjects participating in this study had a median BMI of 21.7 [interquartile range (IQR): 21.3, 22.7] kg/m^2^ in the ND lean group, 31.50 [31.15, 31.70] kg/m^2^ in the ND with obesity group, and 32.4 [IQR: 29.3, 32.9] kg/m^2^ in the T2DM with obesity group (Table 1, median [IQR], *P* < 0.05). Other clinical characteristics and adipose tissue parameters are shown in Table 1.

### 3.2. Abdominal Adipose Tissue miRNAs Are Elevated in People with Obesity and Diabetes

**Figure 1:**
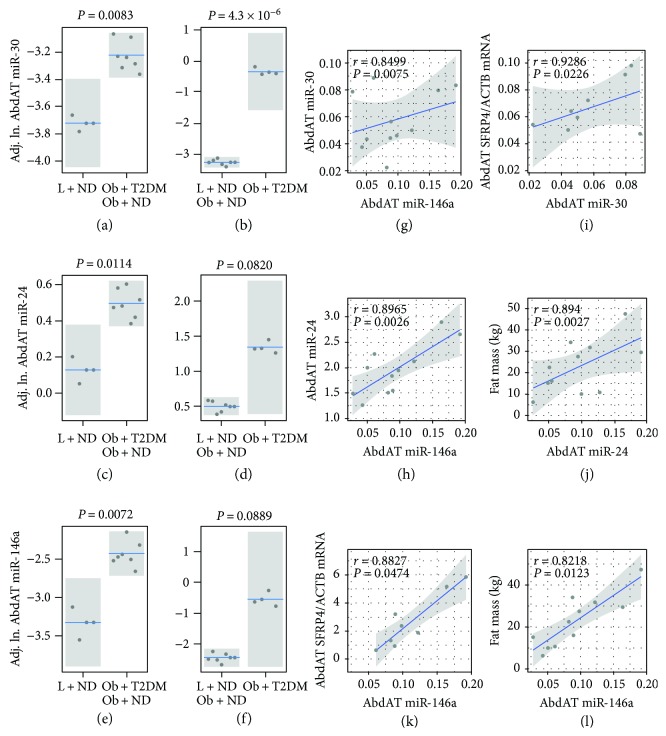
Differential expression and correlation analysis identify key coexpressed abdominal adipose tissue (AbdAT) miRNAs that appear to interact with AbdAT SFRP4. AbdAT miRNAs measured by qRT-PCR are elevated in subjects with obesity and diabetes (a–f). Levels of select miRNAs, mRNAs, and proteins in AbdAT were modeled using generalized linear models (GLMs with a gamma family and log link) as a function of a T2DM-related variable with two levels (ND, T2DM) and a BMI-related variable with two levels (lean, obesity). GLMs controlled for potential confounders including age, gender, and ethnicity. Visualization of the regression models was rendered with the R package *visreg*. Boxplots with overlapped dot plots display the adjusted values (partial residuals). Mean represented by the blue line and 95% confidence interval of the mean as a grey band. ND: subjects without type 2 diabetes; T2DM: subjects with type 2 diabetes; Adj. ln: natural logarithm value of the measurement adjusted for confounders including age, gender, and ethnicity. Partial correlation analysis identified seemingly coregulated miRNAs and significant associations between specific AbdAT miRNAs and AbdAT SFRP4 and measures of adiposity (g–l). Correlation scatterplots including miRNA variables display the −ΔCt data from differentially expressed AbdAT miRNAs and were generated with the R package *ggplot2*. The reported partial correlation that controlled for age, gender, and ethnicity was calculated with the *ppcor* package. The blue line and gray band represent the linear fit of the plotted values and the 95% confidence interval of the fit, respectively. The secondary analysis of miRNA expression in abdominal adipose tissue was performed in seventeen subjects (lean *N* = 7 and subjects with obesity *N* = 10), as per sample availability.

To assess the role of AbdAT miRNAs in the context of obesity independently from T2DM and vice versa, we performed miRNA profiling in the specific tissue and implemented generalized linear modeling controlling for the potential confounding effects of age, gender, and ethnicity. We found that AbdAT miR-30d is significantly elevated in people with obesity (as compared to ND lean controls, *P* = 0.0083—the “obesity context” comparison, Figure 1(a)) and in T2DM subjects (as compared to ND lean and ND with obesity controls, *P* = 4.3 × 10^−6^—the “T2DM context” comparison Figure 1(b)). Similarly, two other miRNAs, namely, miR-24 and miR-146a, were found significantly elevated in the AbdAT in the “obesity context” (*P* = 0.0114 and *P* = 0.0072, respectively, Figures 1(c) and 1(e)) and marginally significantly (*P* < 0.1) elevated in the same tissue in the “T2DM context” (Figures 1(d) and 1(f)). Notably, these three AbdAT miRNAs were found highly correlated among themselves (r_miR.30d~miR.146a_ = 0.85 and r_miR.24~miR.146a_ = 0.90, both with *P* < 0.01, Figures 1(g) and 1(h)).

### 3.3. Abdominal Adipose Tissue Levels of miR-30d and miR-146a Positively Correlate with the Antiangiogenic Factor SFRP4 in the Same Tissue

AbdAT miR-30d and miR-146a positively and significantly correlated with AbdAT expression levels of SFRP4 (r_miR.30d~SFRP4_ = 0.93 and r_miR.146a~SFRP4_ = 0.88, both with *P* < 0.05, Figures 1(i) and 1(k)). Consistent with its reported antiangiogenic functions, transcript levels of SFRP4 in the fat tissue negatively and significantly correlated with the respective tissue capillary density [11]. However, we did not detect significant correlations between AbdAT SFRP4 [11] or the differentially expressed AbdAT miRNAs (this study) with VEGF transcript levels in the fat tissue.

### 3.4. Abdominal Adipose Tissue Levels of miR-24 and miR-146a Positively Correlate with Measures of Adiposity

**Figure 2:**
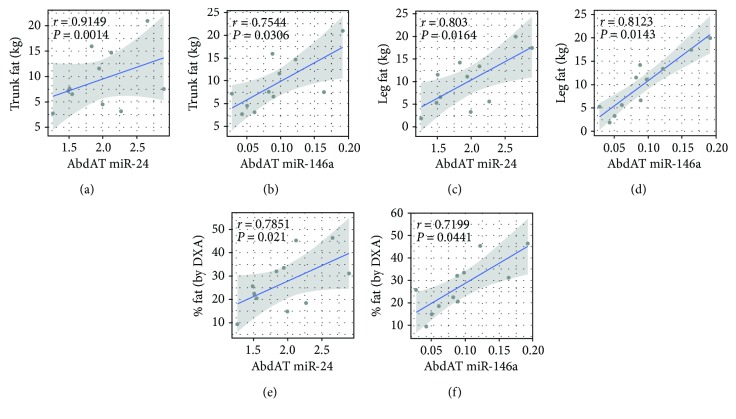
Additional measures of adiposity correlate with abdominal adipose tissue (AbdAT) miR-24 and miR-146a. Partial correlation analysis identified significant associations between specific AbdAT miRNAs and measures of adiposity (a–f). Correlation scatterplots display the −ΔCt data from differentially expressed AbdAT miRNAs and were generated with the R package *ggplot2*. The reported partial correlation that controlled for age, gender, and ethnicity was calculated with the *ppcor* package. The blue line and gray band represent the linear fit of the plotted values and the 95% confidence interval of the fit, respectively.

AbdAT miR-24 and miR-146a significantly correlated with measures of adiposity including fat mass (r_miR.24~fat.mass_ = 0.894, r_miR.146a~fat.mass_ = 0.822, both *P* < 0.05, Figures 1(j) and 1(l)), trunk fat (r_miR.24~trunk.fat_ = 0.91 and r_miR.146~trunk.fat_ = 0.75, both *P* < 0.05, Figures 2(a) and 2(b)), leg fat (r_miR.24~leg.fat_ = 0.80 and r_miR.146a~leg.fat_ = 0.81, both *P* < 0.02, Figures 2(c) and 2(d)), and % fat by DXA (r_miR.24~%.fat_ = 0.78 and r_miR.146a~%.fat_ = 0.72, both *P* < 0.05, Figures 2(e) and 2(f)).

## 4. Discussion

miRNAs influence the expression of target genes in multiple ways: directly, by binding to target gene transcripts to alter (usually reduce) mRNA transcript levels; indirectly, for example, by altering the expression of transcription factors, which, in turn, regulates the expression of target genes; and by acting on cofactors of transcription factors or on genes in signaling networks that control the expression of specific target genes/proteins. Target proteins, in turn, can function to regulate feedback loops that control the expression of miRNAs [22].

This study uncovers a potential regulatory link between AbdAT miRNAs and SFRP4 and supports our previous finding that AbdAT is a major contributor of circulating SFRP4 and that SFRP4 plays important roles in adipose tissue and T2DM pathophysiology [11, 16]. In the study by Garufi et al. [11], we reported that circulating SFRP4 protein abundance significantly correlated with the levels of AbdAT SFRP4 mRNA (*r* = 0.60, *P* = 0.029) and negatively correlated with the AbdAT vascularization as measured by capillary density (*r* = −0.65, *P* < 0.05). We also reported that circulating SFRP4 protein levels correlated with AbdAT VEGF mRNA (*r* = −0.67, *P* < 0.05) and with AbdAT local inflammation as measured by secreted MIP1*α* (*r* = 0.74, *P* < 0.05) [11]. Similarly, we found that AbdAT-SFRP4 was also negatively associated with the AbdAT capillary density (*r* = −0.71, *P* < 0.05) [11]. Importantly, it was previously reported that the levels of circulating SFRP4 negatively correlated with whole body insulin sensitivity and the insulin capacity to suppress lipolysis [11]. Altogether, these findings suggested that SFRP4 might work in a paracrine and endocrine manner to modulate AbdAT capillarity and systemic insulin sensitivity, respectively.

Now, using samples from the same parent study cohort, we have uncovered new evidence that supports the existence of obesity- and T2DM-related mechanisms that upregulate the production of the secreted antiangiogenic factor SFRP4 in the AbdAT, in close association with the expression of fat tissue-derived miRNAs (specifically miR-30d, miR-146a). Interestingly, the differentially abundant levels of miR-146a in the fat tissue of subjects with obesity and diabetes, and its positive correlation with the level of SFRP4 mRNA in the same tissue suggest the existence of a negative feedback loop between these two molecules, as they are reported to impact opposite effects on angiogenesis [11, 30]. Supporting our reasoning, the miRNA target prediction tool miRanda identified SFRP4 as a target of miR-146a. This implies that in AbdAT of subjects with obesity and T2DM, the elevation of SFRP4 levels may induce the expression of miR-146a as a feedback regulatory loop to self-limit the potentially detrimental overexpression of SFRP4. Consequently, this loop may contribute to fine-tuning the remodeling of the adipose tissue landscape (e.g., capillary microvasculature).

On the other hand, fat cell enlargement was previously reported as an independent marker of insulin resistance and hyperleptinaemia [31], and we previously found that circulating SFRP4 protein correlated with measures of adiposity such as body fat and BMI [11]. We now report that adipose tissue-expressed miRNAs miR-24, miR-30d, and miR-146a also significantly associate with measures of adiposity such as whole body, trunk, and leg fat mass as well as percent body fat (measured by DXA). Remarkably, our published studies profiling circulating miRNAs in independent cohorts covering the full diabetes spectrum [including prediabetes, T2DM, latent autoimmune diabetes of adults (LADA), and type 1 diabetes] have identified these three miRNAs among characteristic signatures that differentially correlate with clinical measures and indices of glycemic control, insulin resistance, and beta cell function and allow for fairly sensitive and specific classification of diabetes subtypes [16, 28].

Taken together, our current and previously published work discussed in this manuscript suggests an important role for the apparently coregulated (in AbdAT) miRNA trio miR-24/miR-30d/miR-146a and SFRP4 in obesity-related pathological events leading to insulin resistance and T2DM. The mechanism through which these molecules exert their effects on obesity is still unclear, but these studies suggest that miRNA-regulated adipose tissue-secreted SFRP4 is related to adiposity and contributes to global insulin resistance in a paracrine and endocrine manner. However, the cross-sectional nature of the study and the limited number of subjects in each cohort limit the generalizability of our conclusions. Because the study is based, at least in part, on the analysis of correlative associations, causal relationships cannot be determined with certainty. Further mechanistic studies are warranted.

## 5. Conclusion

We show here, for the first time to our knowledge, that correlated levels of miR-24, miR-30d, and miR-146a are elevated in AbdAT from people with obesity and diabetes. The positive correlations between the levels of miR-24, miR-146a, and miR-30d and the levels of SFRP4 transcripts in adipose tissue suggest regulatory loops between these molecules. The correlations between the levels of specific miRNAs and SFRP4 in AbdAT and measures of whole body adiposity (detected in this study) together with previous findings in the same cohort demonstrating associations among AbdAT SFRP4, circulating SFRP4, AbdAT capillarity, and whole body insulin sensitivity [11] suggest a role for abdominal fat and fat-derived miRNA-regulated SFRP4 in the modulation of systemic insulin sensitivity/resistance. Collectively, our findings suggest novel roles for miRNAs in the regulation of mechanisms that impact adiposity and remodeling of the adipose tissue landscape, as well as mechanisms that impact insulin resistance in obesity and T2DM.

## Figures and Tables

**Table 1:** Demographic and clinical characteristics of the study cohorts.

	Level	ND lean (*N* = 7)	ND with obesity (*N* = 5)	T2DM with obesity (*N* = 5)	*P*
Age (median [IQR])		21 [20, 23]	26 [22, 27]	52 [51, 56]	0.004
Gender (%)	F	2 (28.6)	1 (20.0)	4 (80.0)	0.106
	M	5 (71.4)	4 (80.0)	1 (20.0)	
Ethnicity (%)	AA	2 (28.6)	3 (60.0)	3 (60.0)	0.636
	Asian	1 (14.3)	0 (0.0)	0 (0.0)	
	Caucasian	4 (57.1)	2 (40.0)	2 (40.0)	
BMI (median [IQR])		21.70 [21.30, 22.70]	31.50 [31.15, 31.70]	32.40 [29.30, 32.90]	0.002
Waist-to-hip ratio (median [IQR])		0.80 [0.75, 0.80]	0.90 [0.90, 0.90]	0.90 [0.90, 0.90]	0.027
% fat (median [IQR])		18.45 [14.80, 22.77]	31.56 [22.51, 32.04]	34.04 [33.45, 45.30]	0.005
VO2 max (ml kg min) (median [IQR])		41.51 [34.46, 41.58]	21.73 [21.18, 22.29]	20.09 [18.03, 22.37]	0.023
GDR FFM (median [IQR])		10.84 [10.12, 12.66]	5.41 [5.09, 7.12]	6.02 [2.84, 6.76]	0.022
Glucose (median [IQR])		88.25 [87.75, 89.38]	87.00 [84.50, 92.00]	130.50 [126.00, 156.50]	0.006
Insulin (median [IQR])		4.28 [3.73, 5.24]	10.60 [10.30, 11.10]	16.10 [12.75, 31.80]	0.007
HOMA-IR (median [IQR])		0.93 [0.80, 1.11]	2.13 [2.07, 2.36]	5.14 [3.40, 11.15]	0.003

Comparison between groups for continuous variables was done using the Wilcoxon test. Comparison for categorical variables was done using the Fisher exact test. Data is presented as median with [interquartile range (IQR)] or as percent (%). ND: subjects without diabetes; T2DM: subjects with type 2 diabetes mellitus; BMI: body mass index; GDR: glucose disposal rate. Percent body fat was measured by DXA. Glucose disposal rate was measured by hyperinsulinemic-euglycemic clamp to calculate insulin resistance. VO2 max was a measure of aerobic exercise capacity. The clinical data and tissue angiogenesis were previously described [5].

## Additional Points

*Study Importance*. What is already known about this subject? (a) There is a strong link between adiposity and insulin resistance and type 2 diabetes (T2DM). (b) Abdominal adipose tissue (AbdAT) is a major contributor to circulating SFRP4. SFRP4 plays important roles (e.g., may inhibit vascularization and promote tissue inflammation) in obesity and T2DM pathophysiology. (c) Circulating levels of miR-24, miR-30d, and miR-146a have been previously involved with adipogenesis, obesity, and T2DM. What does this study add? (a) AbdAT levels of miR-24, miR-30d, and miR-146a are elevated in subjects with obesity and T2DM and positively correlate with the level of SFRP4 transcripts in the same tissue. Notably, SFRP4 and miR-146a display opposite antiangiogenic and angiogenic activities, respectively. This suggests a negative feedback loop between miR-146a and SFRP4 in human AbdAT. (b) These results emphasize the roles of AbdAT miRNAs (i.e., miR-24, miR-30d, and miR-146a) and SFRP4 in regulating mechanisms that govern AbdAT remodeling and systemic insulin sensitivity in obesity and T2DM.
